# Higher levels of disease-related knowledge reduce medical acceleration in patients with inflammatory bowel disease

**DOI:** 10.1371/journal.pone.0233654

**Published:** 2020-06-05

**Authors:** Jihye Park, Hyuk Yoon, Cheol Min Shin, Young Soo Park, Nayoung Kim, Dong Ho Lee

**Affiliations:** Department of Internal Medicine, Seoul National University Bundang Hospital, Seongnam, Korea; McMaster University, CANADA

## Abstract

**Background and aims:**

The disease-related knowledge levels in patients with inflammatory bowel disease (IBD) are important because it could affect the self-management ability and adaptive coping strategies. We set out to determine whether higher levels of disease-related knowledge reduce medical acceleration.

**Methods:**

We evaluated the levels of disease-related knowledge in all patients at the time of enrollment for SNUBH IBD cohort using the validated IBD-KNOW questionnaire. Clinical data were prospectively collected and the factors related to step-up therapy were analyzed. Step-up therapy was defined as the new use of corticosteroids, immunomodulators, or biologics after the enrollment.

**Results:**

Between April 2017 and January 2019, 298 patients were enrolled (mean age, 39.8 years; males, 69.5%); 193 patients (64.8%) had ulcerative colitis and 105 (35.2%) had Crohn’s disease. The mean disease duration was 35.8 months. During the mean follow-up of 14.7 months, 90 patients (30.2%) underwent step-up therapy and 208 (69.8%) underwent continuous therapy. The prevalence of continuous therapy increased with increasing IBD-KNOW scores (*p* for trend = 0.019). Cox proportional hazards analysis revealed that high IBD-KNOW scores (≥ 16) (hazards ratio [HR]: 0.498, 95% confidence interval [CI]: 0.276–0.897, *p* = 0.020) was negatively associated with the step-up therapy.

**Conclusions:**

Higher disease-related knowledge could reduce the requirement of step-up therapy in IBD. The IBD-KNOW score was independently predictive of step-up therapy.

## Introduction

Inflammatory bowel disease (IBD) has become a global disease affecting 3.5 million people with accelerating incidence in newly industrialized countries including the Republic of Korea. [1, 2] The medical therapy for IBD involves different drug categories, including 5-aminosalicylates (5-ASA), corticosteroids, immunomodulators, and biologics. Step-up therapy for IBD is an approach of adding or changing therapies if first-line or less toxic approaches are unsuccessful within an appropriate period. [3] The Korean National Health Insurance coverage policy supports only step-up therapy. Biologics can be prescribed for patients with moderate to severe disease who are unresponsive or intolerable to conventional therapeutic agents such as 5-ASA, corticosteroids, or immunomodulators. [4, 5] Moreover, the health care costs have increased recently due to the rising medical expenditures driven by the increasing use of biologics. [6] Therefore, medical acceleration could be a meaningful clinical outcome related to disease progression.

Disease-related knowledge levels of IBD patients are important because of being related to the self-management ability of patients. [7] Greater IBD knowledge levels were positively associated with adaptive coping strategies in a previous study. [8] Further, higher levels of knowledge of the disease seem to positively impact the adherence and patient outcomes in IBD. [9, 10] A measurement questionnaire of disease-related knowledge in IBD, the Crohn’s and Colitis Knowledge (CCKNOW) score, was developed by *Eaden* et al. in 1999 in the United Kingdom and has been used widely. [11] However, CCKNOW is not indicative of updated knowledge such as that regarding biologic therapy and has several limitations such as the uneven distribution of disease-specific items and a higher correct answer ratio of “true.” Therefore, a novel disease-related knowledge score, the Inflammatory Bowel Disease Knowledge (IBD-KNOW) score, was developed and validated. [12] Recently, the usefulness of the IBD-KNOW questionnaire was identified in both the United States and Korean patients. [13] Several studies have investigated the independent risk factors of a poor IBD-KNOW score. [14, 15] However, to our knowledge, the association between disease-related knowledge levels of IBD and clinical outcomes such as medical acceleration has not been studied so far. Therefore, we set out to determine whether higher levels of disease-related knowledge regarding IBD reduce medical acceleration in patients using the IBD-KNOW questionnaire.

## Methods

### Patients

From April 2017, we initiated the set-up of the IBD cohort and enrolled all patients who visited the IBD clinic at Seoul National University Bundang Hospital (Seongnam, Republic of Korea). The exclusion criteria were: (i) less than 16 years of age and (ii) patients who declined to participate in the study. For the enrolled patients, extensive medical data were collected every time they visited our clinic using electronic medical records. For this study, we additionally excluded patients who were followed-up for lesser than 12 months from the enrollment. This study was approved by the Institutional Review Board of Seoul National University Bundang Hospital (IRB No: B-1702/382-003). Written informed consent for the use of medical records was provided by all participants.

### Data collection and definitions

The IBD-KNOW questionnaire, which comprises 24 items quantifying the knowledge of various aspects of IBD (anatomy, function, epidemiology, diet/lifestyle, general knowledge, medication, complication, surgery, reproduction, and vaccination), was distributed to all patients at the time of enrollment (S1 Appendix). We reviewed the electric medical records of these patients, and baseline clinical characteristics, including date of birth, sex, cigarette smoking status (past and current), date of diagnosis, comorbid disease, IBD subtype, history of IBD-related surgery, hospitalization, and emergency room visit, and previous and current medications, were acquired. The “medication history” was defined to include both drugs that were being used at the beginning of the monitoring period and those had been used before. We calculated the IBD-KNOW total score and defined the group with high levels of disease-related knowledge group as patients received 16 points or more (approximately the top 20% of the entire cohort).

Step-up therapy was defined as meeting any of the following criteria: new use of (i) systemic corticosteroids, (ii) immunomodulators including azathioprine, 6-mercaptopurine, methotrexate, and cyclosporine, or (iii) biologics including anti-tumor necrosis factor-alpha (TNF-α) and anti-α4β7-integrin agents associated with deterioration in clinical symptoms, endoscopic inflammation, or serologic parameters after the enrollment. [3, 16] Also, the biologics dose escalation is defined as step-up therapy. Continuous therapy was defined as not having step-up therapy, which included maintaining or stopping current therapy.

### Statistical analysis

Means and standard deviations or medians and ranges were calculated for all continuous variables, as appropriate. Categorical variables were expressed as proportions (%) and statistical analyses were performed to compare the groups of variables. Student’s t tests (Mann-Whitney U tests) were used for continuous variables and the chi-square tests (Fisher’s exact tests) or linear-by linear association tests were used for categorical variables. Kaplan-Meier analyses (log-rank tests) were carried out to compare the cumulative risk of medical acceleration associated with the IBD-KNOW score categories, previous corticosteroids use, and previous biologics use. Cox proportional hazards analyses were conducted to evaluate the independent risk factors of cumulative medical acceleration with adjustment for various confounders. Covariates with a *p-*value of < 0.1 in the univariate analysis were subjected to multivariable Cox proportional hazards analysis. All statistical analyses were performed using the Statistical Package for Social Sciences (SPSS version 25.0; SPSS Inc., Armonk, NY, USA). A *p-*value of < 0.05 was considered statistically significant. The statistical methods of this study were reviewed by the Medical Research Collaborating Center at Seoul National University Bundang Hospital.

## Results

### Patient baseline characteristics

Between April 2017 and January 2019, 298 patients who visited the IBD clinic of Seoul National University Bundang Hospital were enrolled. The mean age was 39.8 ± 15.5 years, and 207 patients (69.5%) were male. Overall, 193 patients (64.8%) were diagnosed with ulcerative colitis (UC) and 105 patients (35.2%) were diagnosed with crohn’s disease (CD). The mean disease duration was 4.0 ± 4.5 years (median, 2.0 years; range, 0–28 years). During the mean follow-up of 14.7 months, 208 patients (69.8%) underwent continuous therapy and 90 patients (30.2%) underwent step-up therapy. The baseline characteristics are summarized in Table 1. The proportion of patients who acquired a higher IBD-KNOW score (≥ 16) was 25.5% in the continuous therapy group and 14.4% in the step-up therapy group (*p* = 0.047). The correct answer rates of each domain of the IBD-KNOW score are listed in S2 Appendix. The domain of diet and lifestyle, general knowledge, and medication resulted in significant differences in the correct answer rates. The proportion of patients who had been previously treated with systemic corticosteroids was 57.7% in the continuous therapy group and 68.9% in the step-up therapy group (*p* = 0.072).

**Table 1:** Baseline characteristics of the study subjects at collecting consent.

Variables	Continuous therapy (N = 208)	Step-up therapy (N = 90)	*p value
Age (years)	39.9 ± 15.0	39.5 ± 16.7	0.847
Males (versus female)	144 (69.6%)	63 (70.0%)	1.000
Smoking			0.102
Never	125 (60.1%)	65 (72.2%)	
Ex-smoker	42 (20.2%)	15 (16.7%)	
Current smoker	41 (19.7%)	10 (11.1%)	
Disease duration (years)	4.0 ± 4.5	3.9 ± 4.5	0.783
Charlson comorbidity index			0.363
0	178 (85.6%)	81 (90.0%)	
1	21 (10.1%)	6 (6.7%)	
≥2	9 (4.3%)	3 (3.3%)	
CD (versus UC)	74 (35.6%)	31 (34.4%)	0.895
Disease extent (UC, N = 193)			0.926
Proctitis	45 (33.6%)	9 (15.3%)	
Left sided colitis	43 (32.1%)	27 (45.8%)	
Pancolitis	46 (34.3%)	23 (39.0%)	
Age at diagnosis (N = 298)			0.926
A1 (≤ 16 years)	8 (3.8%)	3 (3.3%)	
A2 (17–40 years)	124 (59.6%)	54 (60.0%)	
A3 (> 40 years)	76 (36.5%)	33 (36.7%)	
Location of disease (CD, N = 105)			0.284
L1 (terminal ileum)	17 (23.0%)	6 (19.4%)	
L2 (colon)	0 (0.0%)	1 (3.2%)	
L3 (ileocolon)	57 (77.0%)	24 (77.4%)	
Behavior of disease (CD, N = 105)			0.331
B1 (nonstricturing nonpenetrating)	36 (48.6%)	20 (64.5%)	
B2 (stricturing)	14 (18.9%)	4 (12.9%)	
B3 (penetrating)	24 (32.4%)	7 (22.6%)	
Perianal disease (CD, N = 105)	30 (40.5%)	14 (45.2%)	0.205
Previous intestinal resection	11 (5.3%)	1 (1.1%)	0.115
Previous hospitalization	82 (39.4%)	35 (38.9%)	1.000
Previous emergency room visit	68 (32.7%)	24 (26.7%)	0.340
Extraintestinal manifestation (≥ 1)	34 (16.3%)	11 (12.2%)	0.481
IBD-KNOW score (≥ 16)	53 (25.5%)	13 (14.4%)	0.047
Medication history			
Corticosteroid	120 (57.7%)	62 (68.9%)	0.072
Immunomodulator	93 (44.7%)	42 (46.7%)	0.800
Biologic	44 (21.2%)	17 (18.9%)	0.755

Variables are expressed as mean ± SD or n (%).

*p value for comparing continuous therapy group and step-up group.

CD, Crohn's disease; UC, ulcerative colitis; TNF, tumor necrosis factor; SD, standard deviation

### Medical acceleration rate according to the IBD-KNOW scores

**Fig 1:**
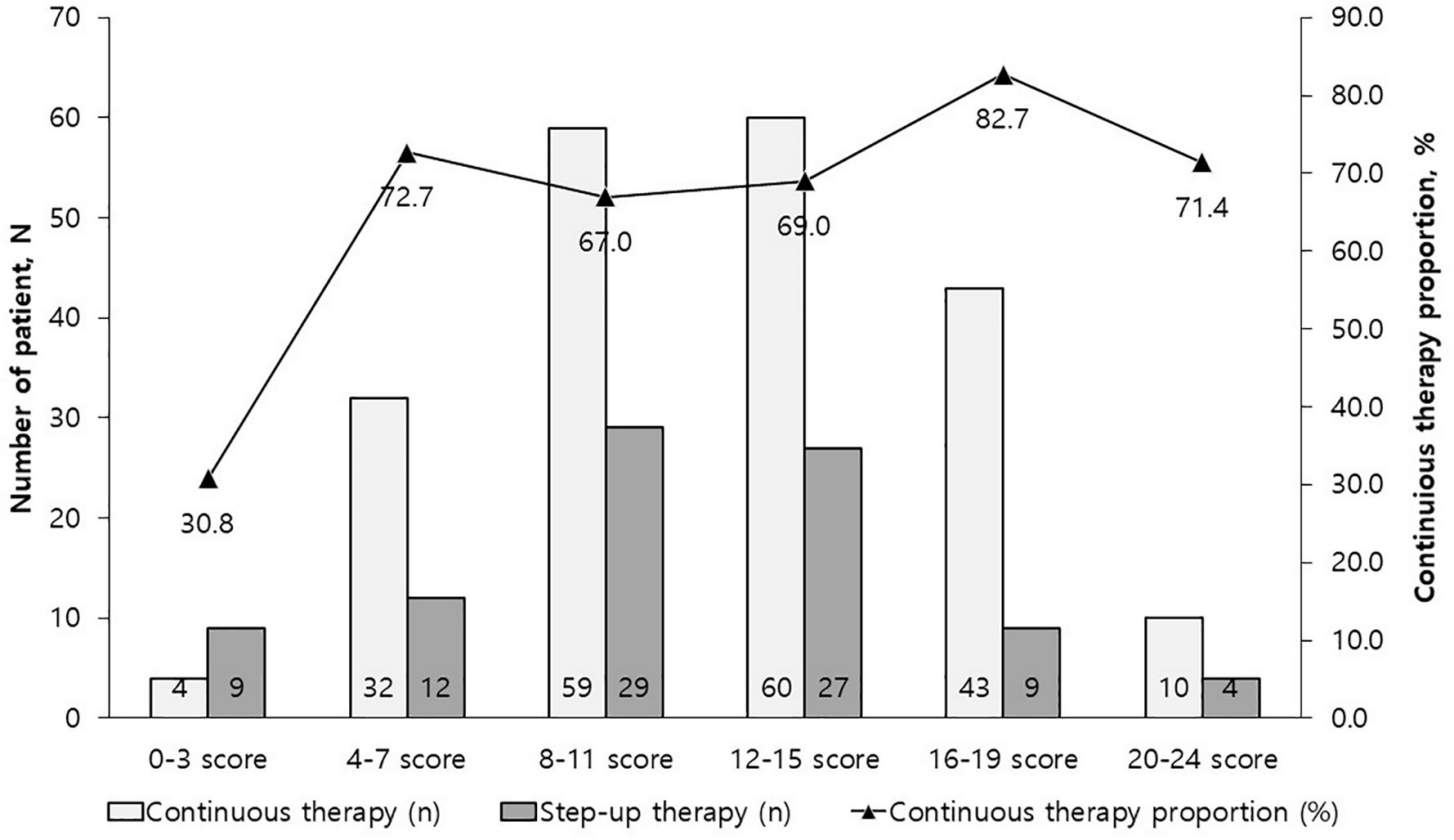
The number and proportion of patients with inflammatory bowel disease on continuous therapy stratified by IBD-KNOW scores. The proportion of patients received continuous therapy was increased with increasing IBD-KNOW score (*p* for trend = 0.019). Continuous therapy was defined as not having step-up therapy, which included maintaining current therapy or stopping current therapy.

The mean IBD-KNOW score was 11.7 ± 4.7 (median, 11; range, 1–23). Of the 298 patients, 66 patients (22.1%) had 16 points or more with a total perfect score of 24. The distributions of the IBD-KNOW score stratified according to 6 subscales (score of 0–3, 4–7, 8–11, 12–15, 16–19, 20–24) are presented in Fig 1. The proportion of patients who received continuous therapy increased with increasing IBD-KNOW scores (*p* for trend = 0.019). As for step-up therapy, the proportion of patients who received immunomodulators decreased with increasing IBD-KNOW scores (*p* for trend = 0.035; Table 2). The proportion of patients who received biologics decreased with increasing IBD-KNOW scores (*p* for trend = 0.012; Table 2).

**Table 2:** The proportion of patients with inflammatory bowel disease on step-up therapy stratified by IBD-KNOW scores.

	0–3 score (N = 13)	4–7 score (N = 44)	8–11 score (N = 88)	12–15 score (N = 87)	16–19 score (N = 52)	20–24 score (N = 14)	*p for trend
Use of corticosteroids	6 (46.2%)	6 (13.6%)	21 (23.9%)	19 (21.8%)	8 (15.4%)	3 (21.4%)	0.317
Initiation of immunomodulators	5 (38.5%)	3 (6.8%)	10 (11.4%)	12 (13.8%)	1 (1.9%)	1 (7.1%)	0.035
Initiation of biologics	5 (38.5%)	6 (13.6%)	9 (10.2%)	10 (11.5%)	4 (7.7%)	0 (0.0%)	0.012

Variables are expressed as n (%).

IBD-KNOW, Inflammatory Bowel Disease Knowledge

### Prognostic factors for medical acceleration

**Fig 2:**
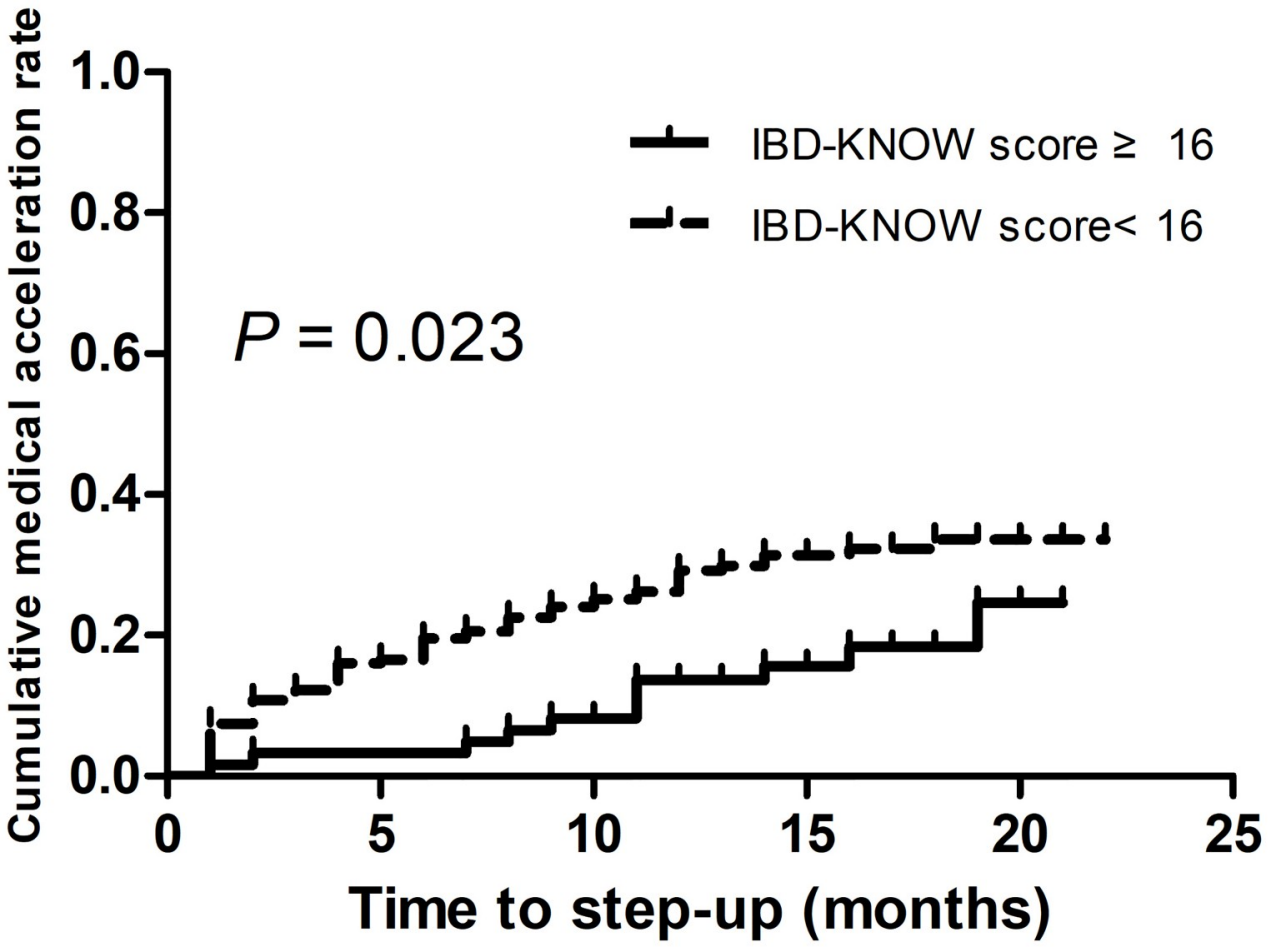
Kaplan-Meier curve showing cumulative medical acceleration rate in relation to IBD-KNOW scores.

Kaplan-Meier analyses (log-rank tests) were carried out to determine the factors associated with medical acceleration in IBD patients. A higher IBD-KNOW score (≥ 16, *p* = 0.023; Fig 2) was found to be significantly associated with medical acceleration. In the Cox proportional hazards analysis, a high IBD-KNOW score (≥ 16) (hazards ratio [HR]: 0.498, 95% confidence interval [CI]: 0.276–0.897, *p* = 0.020) was negatively associated with medical acceleration (Table 3).

**Table 3:** Cox proportional hazards analysis for medical acceleration in inflammatory bowel disease.

Variables	Univariate analysis			Multivariate analysis		
	HR	95% CI	*p value	HR	95% CI	*p value
Age (≥ 40 years)	0.952	0.628–1.442	0.815			
Males	0.967	0.616–1.518	0.883			
Smoking						
Never	1.000	reference	0.129			
Ex-smoker	0.734	0.419–1.288	0.281			
Current smoker	0.531	0.273–1.034	0.063			
Disease duration (≥ 5 years)	1.094	0.703–1.702	0.691			
Charlson comorbidity index						
0	1.000	reference	0.726			
1	0.727	0.317–1.666	0.451			
≥2	0.834	0.263–2.642	0.758			
CD versus UC	0.878	0.568–1.356	0.557			
Previous intestinal resection	0.218	0.030–1.566	0.130			
Previous hospitalization	0.882	0.577–1.347	0.560			
Previous emergency room visit	0.711	0.446–1.136	0.154			
Extraintestinal manifestation (≥ 1)	0.718	0.382–1.349	0.303			
**IBD-KNOW score (≥ 16)**	**0.506**	**0.281–0.912**	**0.023**	**0.498**	**0.276–0.897**	**0.020**
Medication history						
**Corticosteroid**	**1.466**	**1.938–2.291**	**0.093**	**1.495**	**0.957–2.337**	**0.077**
Immunomodulator	0.954	0.631–1.445	0.826			
Biologics	0.841	0.496–1.427	0.520			

*p value for comparing patients with step-up therapy and patients with continuous therapy

HR, hazard ratio; CI, confidence interval; CD, Crohn's disease; UC, ulcerative colitis

## Discussion

The disease-related knowledge levels in patients with IBD are important because it could affect the self-management ability and adaptive coping strategies among such patients. [7, 8] *Colombara* et al. demonstrated that the knowledge levels of IBD patients have a strong potential to help in cost reduction. [17] However, there are very few studies on the association between knowledge levels and clinical outcomes. *Eaden* et al. studied the relationship between knowledge about UC and the risk of developing colorectal cancer, but no evidence to support this relationship was demonstrated. [18] In addition, *Hou* et al. assessed the correlation between the patient’s knowledge and health-related quality of life, but no significant correlation between the overall CCKNOW and short inflammatory bowel disease questionnaire (SIBDQ) scores was noted. [14] To our knowledge, our study was the first to evaluate the relationship between the knowledge levels regarding IBD and medical acceleration. We demonstrated that a higher knowledge level of IBD could affect the prevalence of continuous therapy rather than that of step-up therapy. Patients with a higher knowledge level regarding IBD were less likely to use biologics and corticosteroids. In addition, we confirmed that the IBD-KNOW score of 16 or more could lower the proportion of medical acceleration by approximately half (HR: 0.499) according to the multivariable Cox proportional hazards analysis.

Proper medical compliance and regular follow up are key factors for the successful management of IBD with a reduction in the frequency of a relapse. Disease-specific knowledge positively impacts adherence to therapy and patient satisfaction with their treatment among those with chronic illnesses including IBD. [9, 10, 19, 20] Moreover, drug compliance had a significant association with disease awareness among patients with IBD. [9, 10] *Van der Have* et al. reported that non-adherence to anti-TNF-α agents is significantly associated with illness perceptions. [21] As higher levels of IBD knowledge may increase medication adherence, we speculate that good adherence might have reduced the prevalence of step-up therapy and increased that of continuous therapy in our study. In the analysis of the correct answer rates of each domain of the IBD-KNOW score, the score of the diet and lifestyle, general knowledge, and medication domain was higher in the continuous therapy group than in the step-up therapy group. These domains may affect to increase self-management ability and adaptive coping strategies in IBD patients.

The education of patients with IBD has been demonstrated to improve disease-related knowledge. *Kennedy* et al. developed a patient-centered guidebook for patients with UC and reported significantly higher knowledge levels in the education group compared with that in the control group. [22] *Waters* et al. designed an educational program for IBD with 3-hour sessions over four consecutive weeks and demonstrated higher knowledge levels and patient satisfaction in the education group compared with that in the control group. [23] *Quan* et al. provided workshops lasting 3 hours for IBD patients and showed that the knowledge of participants improved and was retained for at least 3 months. [24] Recently, various strategies such as telemonitoring, tele-education, and teleconsultation have been implemented to reduce disease burden. [25, 26] Therefore, a structured education component could be expected to improve patient knowledge and adherence and decrease medical acceleration including the use of corticosteroids and biologics. Because the patients who earned 16 points or more in the IBD-KNOW score had 50% less risk of the medical acceleration, this point could be a target of the patient education program.

The use of step-up therapy was the primary endpoint in our study as it represents patients who were unresponsive or intolerable to previous medication according to the National Health Insurance coverage guidelines in Korea. Further, medical acceleration is an important endpoint because it is related to a high economic burden. Total health care expenditures nearly doubled between 2006 and 2016, and this was largely due to an annual increase in the average pharmaceutical expenditure. [6] Particularly, the increased usage of biologics has been accompanied by an increase in the crude mean expenditures over time. [27] Conversely, hospitalization and surgery accounted only for a minor part of the health care costs. [28] In our study, higher knowledge levels of IBD were associated with a reduced prevalence of medical acceleration. Therefore, a higher disease-related knowledge level is expected to reduce the health care cost associated with IBD, and this conclusion is consistent with the previous study conducted by *Colombara* et al. [17]

The CCKNOW score has been widely used to measure disease-related knowledge among IBD patients. However, since it was developed a long time ago, the CCKNOW does not comprise questions about biologics. Therefore, as the CCKNOW was inappropriate to investigate the relationship between disease-related knowledge and medical acceleration, we employed the IBD-KNOW questionnaire, which is an updated measurement tool regarding several items including biologics and vaccination. [12] We thus also hope that further studies will now be conducted using the IBD-KNOW.

This study has several limitations that need to be acknowledged. First, the assessment of disease-related knowledge was conducted at only a single time-point during the disease course and was not performed at the time of diagnosis. Serial assessment of the IBD-KNOW score and an assessment at diagnosis would provide more information. However, knowledge levels regarding IBD were shown to be independent of the disease duration in a previous study and our analysis. [11, 12] Another limitation was that we employed a relatively short follow-up duration and could not evaluate the long-term outcomes associated with medical acceleration. Further studies with longer follow-up durations and more events will be needed. Third, drug compliance and adherence were not investigated in this study. Further studies with medical compliance will be needed to make the conclusions more plausible. Fourth, the average disease duration of our IBD cohort was relatively shorter. Further studies with patients who have had long disease duration will be required to confirm the results.

In conclusion, higher levels of disease-related knowledge could reduce medical acceleration in patients with IBD. Structured education programs could help improve patients’ knowledge, enhance patients’ adherence to therapy, and decrease medical acceleration.

## Supporting information

S1 Appendix International version of inflammatory bowel disease knowledge (IBD-KNOW). (DOCX)

S2 Appendix The correct answer rates of each domain of IBD-KNOW score. (DOCX)
