# Supplementary material for: Higher levels of disease-related knowledge reduce medical acceleration in patients with inflammatory bowel disease
Source: PLoS One. 2020 Jun 5;15(6):e0233654. doi: 10.1371/journal.pone.0233654 (PMC7274391; doi:10.1371/journal.pone.0233654)
Supplement: S1 Appendix — (DOCX) [file pone.0233654.s001.docx]

**Appendix 1. International version of inflammatory bowel disease knowledge (IBD-KNOW)**

1. The terminal ileum is the last part of the small bowel. It is located in the right lower abdomen.

a) True

b) False

c) Don’t know

2. The rectum is part of the colon. It starts approximately 15 cm from the anus and finishes at the anus.

.

a) True

b) False

c) Don’t know

3. The function of the colon is to absorb nutrients.

a) True

b) False

c) Don’t know

4. People can survive without the colon, but not without the small bowel.

a) True

b) False

c) Don’t know

5. Specific foods to be avoided in inflammatory bowel disease are well known.

a) True

b) False

c) Don’t know

6. Smoking cessation is important to prevent worsening of Crohn’s disease.

a) True

b) False

c) Don’t know

7. Risk of inflammatory bowel disease increases with family history of this condition.

a) True

b) False

c) Don’t know

8. Inflammatory bowel disease can develop in all age groups, but is more frequent at younger ages.

a) True

b) False

c) Don’t know

9. Anemia may develop if severe inflammation persists.

a) True

b) False

c) Don’t know

10. Crohn’s disease can occur anywhere in the digestive tract, from the mouth to the anus.

a) True

b) False

c) Don’t know

11. Ulcerative colitis rarely involves the rectum.

a) True

b) False

c) Don’t know

12. Inflammatory bowel disease can involve organs other than the bowels.

a) True

b) False

c) Don’t know

13. Inflammatory bowel disease is considered cured if symptoms do not recur after a few years.

a) True

b) False

c) Don’t know

14. Inflammation in the bowels may persist even if the symptoms improve after treatment initiation.

a) True

b) False

c) Don’t know

15. Long-term steroid administration is advised to reduce inflammation recurrence.

a) True

b) False

c) Don’t know

16. Constant blood monitoring is indicated for patients who are on immunosuppressive agents, such as azathioprine, because their white blood cell count may decrease.

a) True

b) False

c) Don’t know

17. Biological agents are mainly used in patients with mild symptoms.

a) True

b) False

c) Don’t know

18. Suppository or enema is used to treat cecal inflammation in patients with ulcerative colitis.

a) True

b) False

c) Don’t know

19. Patients with inflammatory bowel disease for 8–10 years should have colorectal cancer screening.

a) True

b) False

c) Don’t know

20. Permanent colostomy is performed if surgery is indicated for patients with ulcerative colitis.

a) True

b) False

c) Don’t know

21. Patients with Crohn’s disease of the small bowel may be cured after surgery.

a) True

b) False

c) Don’t know

22. Patients with inflammatory bowel disease should stop all the medications when considering pregnancy.

a) True

b) False

c) Don’t know

23. Most patients with inflammatory bowel disease are advised cesarean section delivery.

a) True

b) False

c) Don’t know

24. Immunocompromised patients with inflammatory bowel disease should avoid any kind of vaccination.

a) True

b) False

c) Don’t know
