# Supplementary material for: Higher levels of disease-related knowledge reduce medical acceleration in patients with inflammatory bowel disease
Source: PLoS One. 2020 Jun 5;15(6):e0233654. doi: 10.1371/journal.pone.0233654 (PMC7274391; doi:10.1371/journal.pone.0233654)
Supplement: S2 Appendix — (DOCX) [file pone.0233654.s002.docx]

**Appendix 2. The correct answer rates of each domain of IBD-KNOW score**

|  | **Continuous therapy (N=208)** | **Step-up therapy (N=90)** | ****p* value** |
| --- | --- | --- | --- |
| **Anatomy** | 52.2 ± 33.1 | 50.0 ± 35.9 | 0.614 |
| **Function** | 35.8 ± 39.0 | 30.0 ± 36.6 | 0.230 |
| **Diet and life style** | 57.7 ± 34.2 | 18.3 ± 33.9 | 0.031 |
| **Epidemiology** | 50.0 ± 37.8 | 43.9 ± 37.3 | 0.199 |
| **General knowledge** | 60.1 ± 27.2 | 52.9 ± 27.9 | 0.039 |
| **Medication** | 54.5 ± 28.7 | 46.7 ± 28.0 | 0.030 |
| **Complication** | 64.4 ± 47.9 | 68.9 ± 46.6 | 0.457 |
| **Surgery** | 40.6 ± 37.4 | 35.6 ± 41.2 | 0.298 |
| **Reproduction** | 19.7 ± 32.5 | 20.6 ± 33.4 | 0.839 |
| **Vaccination** | 63.9 ± 48.1 | 52.2 ± 50.2 | 0.058 |
| Variables are expressed as mean ± SD. | | | |
| **p* value for comparing continuous therapy group and step-up group. | | | |
|  | | |  |
